# Immunoproteomic analysis of bacterial proteins of *Actinobacillus pleuropneumoniae *serotype 1

**DOI:** 10.1186/1477-5956-9-32

**Published:** 2011-06-26

**Authors:** Wei Zhang, Jing Shao, Guangjin Liu, Fang Tang, Yan Lu, Zhipeng Zhai, Yang Wang, Zongfu Wu, Huochun Yao, Chengping Lu

**Affiliations:** 1 Key Laboratory of Animal Disease Diagnostic & Immunology, Ministry of Agriculture, Nanjing Agricultural University, Nanjing 210095, China

## Abstract

**Background:**

*Actinobacillus pleuropneumoniae *(APP) is one of the most important swine pathogens worldwide. Identification and characterization of novel antigenic APP vaccine candidates are underway. In the present study, we use an immunoproteomic approach to identify APP protein antigens that may elicit an immune response in serotype 1 naturally infected swine and serotype 1 virulent strain S259-immunized rabbits.

**Results:**

Proteins from total cell lysates of serotype 1 APP were separated by two-dimensional electrophoresis (2DE). Western blot analysis revealed 21 immunoreactive protein spots separated in the pH 4-7 range and 4 spots in the pH 7-11 range with the convalescent sera from swine; we found 5 immunoreactive protein spots that separated in the pH 4-7 range and 2 in the pH 7-11 range with hyperimmune sera from S259-immunized rabbits. The proteins included the known antigens ApxIIA, protective surface antigen D15, outer membrane proteins P5, subunit NqrA. The remaining antigens are being reported as immunoreactive proteins in APP for the first time, to our knowledge.

**Conclusions:**

We identified a total of 42 immunoreactive proteins of the APP serotype 1 virulent strain S259 which represented 32 different proteins, including some novel immunoreactive factors which could be researched as vaccine candidates.

## Background

*Actinobacillus pleuropneumoniae *(APP) is one of the most important swine pathogens worldwide. Of the 15 known APP serotypes, serotype 1 is frequently associated with a range of lung diseases, including fibrinous, hemorrhagic pneumonia, and necrotizing pneumonia. High mortality has been reported in acutely infected pigs and persistent lung lesions have been observed in chronically infected pigs [1].

To control APP pathogenesis, several types of vaccines have been developed that offer various degrees of protection. The traditional chemically-[2,3], genetically-, and irradiation-inactivated [4] whole cell vaccines used thus far have been shown to provide protection against a homologous challenge from APP, but were ineffective in staving off infection by different serotypes.

Some cross-serovar protection has been achieved with live-attenuated vaccines of mutant field isolates, such as mutant strains of APP serovar 7 [5] and serovar 1 [6,7]. Further development of cross-serovar vaccines would benefit from a molecular understanding of APP pathogenesis, which is a complex process involving a number of different potential virulence factors. The most commonly associated virulence factors [8] i.e., ApxI, ApxII, ApxIII, and ApxIV, have been tested as subunit vaccine candidates offering potential cross-serovar protection [9,10]. DNA vaccines encoding multiple Apx toxins offer a novel strategy for protecting against APP infection [11]. In addition to Apx toxins, several other APP proteins have been researched as vaccine candidates, such as the 48 kDa outer membrane protein [12] and the 30 kDa membrane protein of the ABC transporter family [13]. Lipopolysaccharide (LPS)-based vaccines are alternative to protein antigens [14].

The identification of novel antigens as candidate vaccines should be accelerated by modern technologies. The complete genomes of APP L20 (Serotype 5b [15]) and JL03 (serotype 3 [16]) have been sequenced, the outer membrane proteome of serotype 5b has also been characterized [17]. In this paper, we use an immunoproteomic approach to identify APP protein antigens that elicit an immune response in serotype 1 naturally infected swine and serotype 1 virulent strain S259-immunized rabbits. This approach, which we have used to study immunogenicity of other bacterial pathogens [18], combines the specificity of antibody detection with the precision of mass spectral analysis [19] for identifying antigenic bacterial proteins.

## Results

### Two-dimensional electrophoresis (2DE) profiles of APP bacterial proteins and western blot analysis

**Figure 1:**
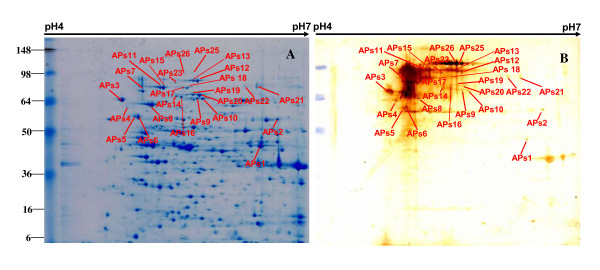
**Comparison of western blot analysis with convalescent sera from swine and duplicated gels of S259 bacterial associated proteins at pH 4-7**. **A**. Coomassie G-250-stained 2DE gel. All identified protein spots were analyzed by MALDI-TOF MS. **B**. Western blot analysis of proteins on 2DE gel as transferred to a PVDF membrane. The primary antibodies were convalescent sera from swine naturally infected with APP serotype 1.

**Figure 2:**
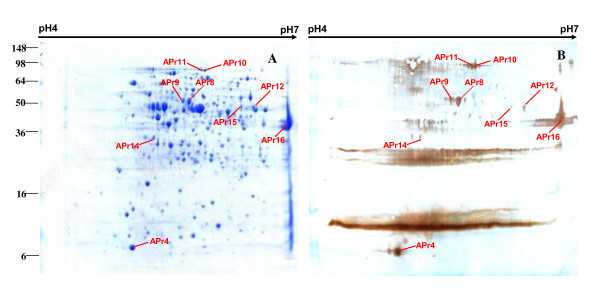
**Comparison of western blot analysis with rabbit hyperimmune sera and duplicated gels of S259 bacterial associated proteins at pH 4-7**. **B. A. **Coomassie G-250-stained 2DE gel. All identified protein spots were analyzed by MALDI-TOF MS. **B**. Western blot analysis of proteins on 2DE gel as transferred to a PVDF membrane. The primary antibodies were hyperimmune sera from S259-immunized rabbits.

**Figure 3:**
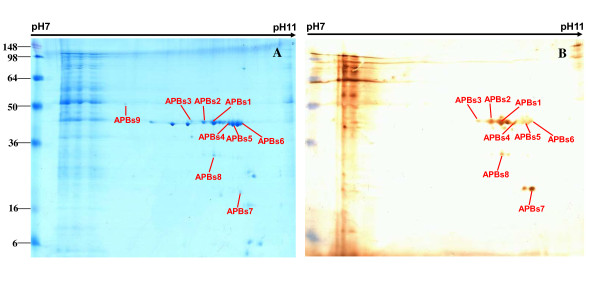
**Comparison of western blot analysis with convalescent sera from swine and duplicated gel of S259 bacterial associated proteins at pH 7-11**. **C. A. **Coomassie G-250-stained 2DE gel. All identified protein spots were analyzed by MALDI-TOF MS. **B**. Western blot analysis of proteins on 2DE gel as transferred to a PVDF membrane. The primary antibodies were convalescent sera from swine naturally infected with APP serotype 1.

**Figure 4:**
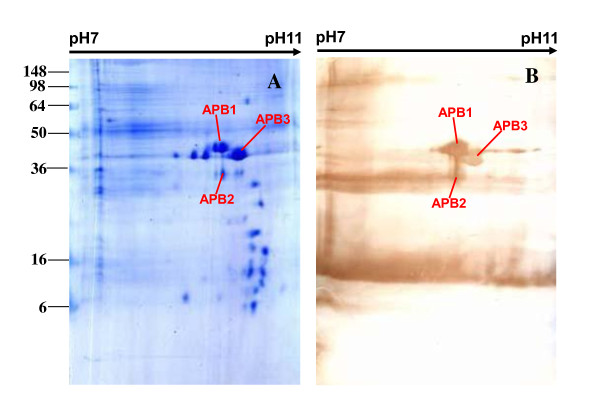
**Comparison of western blot analysis with rabbit hyperimmune sera and duplicated gels of S259 bacterial associated proteins at pH 7-11**. **D. A. **Coomassie G-250-stained 2DE gel. All identified protein spots were analyzed by MALDI-TOF MS. **B**. Western blot analysis of proteins on 2DE gel as transferred to a PVDF membrane. The primary antibodies were hyperimmune sera from S259-immunized rabbits.

Proteins from total cell lysates of APP serotype 1 were separated by 2DE. Two-dimensional separation profiles are shown for separation by isoelectric point (p*I*) in the first dimension over a pH ranges of pH 4-7 (Figures 1A, 2A) and pH 7-11 (Figures 3A, 4A). The separation profiles were highly reproducible in 2DE experiments conducted in triplicate followed by membrane transfer and developing, yielding similar patterns of total proteins and immunoreactive proteins. Figures 1B, 2B, 3B and 4B show the western blot analysis with the convalescent sera from naturally infected APP serotype 1 swine and from hyperimmune sera from S259-immunized rabbit. No specific immunoreactive protein spots were observed when negative control sera were used.

### Alignment of total protein and immunoreactive protein images

Comparisons between 2DE gels and western blot membranes were very difficult to make because only a portion of the proteins on the transferred membrane were immunoreactive and the sensitivity of the western blot was higher than that of Coomassie brilliant blue. In this study, we overcame this challenge by using the layer function of PhotoshopCS to compare western blot and 2DE gels with the ponceaus S stain as an intermediate state. The same membranes used for western blots were also stained with Ponceau S to visualize all proteins. Digital images of the western blots and Ponceau S-stained membranes were compared with the 2DE gels by aligning the images with the layer function of Photoshop CS (Additional file 1, Figure S1, Additional file 2, Figure S2, Additional file 3, Figure S3, Additional file 4, Figure S4). Using this method to align developed membranes and 2DE gels in the pH 4-7 range (Figures 1, 2) and the pH 7-11 range (Figures 3, 4), we found 42 immunoreactive protein spots that represented 32 different proteins. The identified spots were labeled on each gel (Figures 1, 2, 3 and 4).

### Identification of immunoreactive proteins

A total of 42 immunoreactive proteins were identified by immunoproteomics. Corresponding spots for immunoreactive proteins were excised from preparative 2DE gels, subjected to tryptic digestion, and analyzed by mass spectrometry and peptide mass fingerprinting (PMF). For conclusive identification of the protein corresponding with each spot, the probability score of the match (Protein scores > 83 were significant), the weight-average molecular weight (MW), p*I*, number of peptide matches, and percentage of the total translated open reading frame (ORF) sequence covered by the peptides were analyzed. Descriptive data for the identified spots are listed in Tables 1 and 2. Only the proteins ranking first in each matching search are shown. Western blot analysis with convalescent sera from swine naturally infected APP serotype 1, led us to identify 32 immunoreactive spots, representing 25 proteins, including 21 that separated in the pH 4-7 range (Figure 1) and 4 that separated in the pH 7-11 range (Figure 3). Western blot analysis with hyperimmune sera raised in an S259-immunized rabbit revealed 10 immunoreactive spots that represented 7 proteins, including 5 that separated in the pH 4-7 range (Figure 2) and 2 that separated in the pH 7-11 range (Figure 4).

**Table 1:** Summary of immunoreactive proteins identified with convalescent sera from swine naturally infected APP serotype 1

Spot No.	Protein No.	Protein Identified	Theoretical MW/pI	Experimental MW/pI	Peptide match (sequence coverage, %)	PSORTb localization	PSORTb Probability^a^	Mowse score^b^
APs1	gi|307245438	Polyribonucleotide nucleotidyltransferase	77.439/4.96	30.6/6.35	41 (48%)	Cytoplasmic	9.97	336

APs2	gi|190150916	ATP-binding protein	24.383/8.52	40.1/6.50	12 (59%)	Unknown		122

APs3	gi|3913235	Chaperonin GroEL (HSP60 family)	57.722/4.90	63.9/4.67	21 (35%)	Cytoplasmic	9.97	197

APs4	gi|126207624	CTP synthetase	60.032/5.70	60.1/4.70	31 (71%)	Cytoplasmic	9.97	424

APs5	gi|190151264	Chaperone protein dnaK	67.899/4.76	43.5/4.74	41 (64%)	Cytoplasmic	9.97	389

APs7	gi|126209221	fumarate hydratase	50.756/6.14	76.2/5.04	23 (43%)	Cytoplasmic	9.97	157

APs8	gi|303252313	bifunctional UDP-sugar hydrolase/5'-nucleotidase periplasmic precursor	61.029/6.31	61.2/5.08	35 (60%)	Periplasmic	9.76	259

APs10	gi|307245892	Transketolase 2	73.746/5.47	64.2/5.56	36 (42%)	Cytoplasmic	9.97	297

APs11	gi|53728830	Translation elongation factors (GTPases)	77.515/5.10	94.6/5.11	41 (67%)	Cytoplasmic	9.97	356

APs12	gi|46143894	TPR repeat	55.365/9.45	97.6/5.54	28 (62%)	Unknown		373

APs13	gi|46143698	Sugar transferases involved in lipopolysaccharide synthesis	44.335/9.35	97.8/5.52	18 (59%)	CytoplasmicMembrane	10	268

APs14	gi|307245832	hypothetical protein appser1_10340	45.695/6.34	71.7/5.20	18 (55%)	Unknown		245

APs15	gi|303253793	Type I restriction enzyme EcoEI R protein	90.370/6.55	94.1/5.14	40 (59%)	Unknown		393

APs16	gi|307244854	GTP-binding protein typA/bipA	68.343/5.24	69.5/5.26	34 (42%)	CytoplasmicMembrane	7.88	284

APs17	gi|307246005	hypothetical protein appser1_12060	95.894/5.38	94.8/5.36	36 (45%)	Cytoplasmic	9.97	322
APs18					43 (54%)			379

APs19	gi|46143487	Asparagine synthetase A	37.397/5.91	76.1/5.53	21 (65%)	Cytoplasmic	10	290

APs20	gi|53729159	BioD-like N-terminal domain of phosphotransacetylase	77.017/5.50	74.9/5.55	40 (52%)	Cytoplasmic	9.97	384

APs21	gi|126207895	protective surface antigen D15 precursor	89.088/6.30	89.1/6.31	36 (47%)	OuterMembrane	10	302

APs22	gi|307245243	Protective surface antigen D15	89.172/6.30	89.2/6.22	21 (28%)	OuterMembrane	10	152

APs23	gi|303252337	DNA topoisomerase III	72.857/9.07	96.9/5.18	40 (72%)	Cytoplasmic	9.97	385

APs25 APs26	gi|60476777	ApxIIA	102.466/5.50	102.4/5.51 102.4/5.24	32 (35%) 13 (14%)	Extracellular	10	314 117

APBs1 APBs2 APBs3 APBs8	gi|307246344	Outer membrane protein P5	39.638/9.24	39.5/9.31 39.5/9.25 39.7/9.18 34.9/9.31	26 (72%) 24 (61%) 22 (55%) 28 (72%)	OuterMembrane	10	243 220 229 278

APBs4 APBs5 APBs6	gi|307246787	Outer membrane protein P5	38.778/9.51	39.2/9.52 39.2/9.56 39.2/9.62	26 (63%) 25 (76%) 30 (68%)	OuterMembrane	10	260 262 289

APBs7	gi|165976709	ABC-type transport system involved in resistance to organic solvents, auxiliary component	23.399/9.65	23.4/9.60	14 (34%)	Unknown		168

APBs9	gi|46143830	Periplasmic component of the Tol biopolymer transport system	44.768/8.97	48.8/8.56	26 (53%)	Periplasmic	9.76	310

**Table 2:** Summary of immunoreactive proteins identified with hyperimmune sera raised in an S259-immunized rabbit

Spot No.	Protein No.	Protein Identified	Theoretical MW/pI	Experimental MW/pI	Peptide match (sequence coverage, %)	PSORTb localization	PSORTb Probability^a^	Mowse score^b^
APr4	gi|32034816	Ribosomal protein L7/L12	12.322/4.72	10.8/4.75	10 (99%)	Unknown		109

APr8 APr9	gi|165977457	putative aldehyde dehydrogenase	54.094/5.48	54.4/5.46 54.5/5.44	13 (29%) 26 (48%)	Cytoplasmic	9.26	122 221

APr10 APr11	gi|165976190	pyruvate dehydrogenase subunit E1	98.894/5.46	98.1/5.50	19 (28%) 45 (53%)	Cytoplasmic	9.97	433 339

Apr12	gi|53728874	Na+-transporting NADH:ubiquinone oxidoreductase, subunit NqrA	48.573/5.91	50.9/5.97	26 (63%)	Cytoplasmic	9.97	305

Apr14	gi|32035558	Transaldolase	34.959/ 5.02	37.7/5.03	37 (62%)	Cytoplasmic	9.97	117

APBr1 APBr2	gi|190150781	outer membrane protein P5 precursor	39.588/9.17	39.6/9.28 35.9/9.28	18 (55%)	OuterMembrane	10	170 209

APBr3	gi|32034275	Outer membrane protein and related peptidoglycan-associated (lipo)proteins	38.708/9.51	38.7/9.53	7 (29%)	OuterMembrane	10	94

(a) Localization predictions based on PSORT server http://www.psort.org/ evaluation. The values are localization probabilities (from 0 to 10).

(b) Mowse score is the score based on the mowse algorithm in Mascot http://www.matrixscience.com. Protein scores > 83 are significant (p < 0.05).

### Bioinformatics analysis

The BLASTX results are shown in Tables 1 and 2. The single top ranking proteins identified for each spot are listed in the tables. All identified proteins were predicted by PSORTb version 3.0 software http://www.psort.org/. Among the protein spots analyzed with convalescent sera from swine, 2 proteins were annotated as periplasmic proteins, 4 as outermembrane proteins, 1 as an extracellular protein, 11 as cytoplasmic proteins, 2 as cytoplasmic membrane proteins, and 5 were unknown. Meanwhile, in protein spots analyzed with hyperimmune sera from rabbit, 2 proteins were predicted as outermembrane proteins, 4 as cytoplasmic proteins, and 1 was unknown.

## Discussion

In this study, a total of 42 immunoreactive spots, representing 32 different proteins from APP S259 were identified by western blot analysis with convalescent sera from swine and hyperimmune sera from rabbits. Four of those proteins, ApxIIA (spot APs25,26), D15 (spot APs21,22), subunit NqrA (spot APr12), outer membrane protein P5 (spot APBs1,2,3,4,5,6,8; APBr1,2 ), have been demonstrated recently as immunogenic proteins in APP JL03 [20].

ApxIIA, which belongs to the RTX family [21], has been reported to be moderately cytotoxic and weakly hemolytic [22]. Chiang et al. designed DNA vaccines that encode ApxIA or ApxIIA. The vaccines elicited humoral immune responses and protective efficacy in mice [11]. D15 (or its precursor) is an essential component of outer membrane biogenesis and outer membrane protein assembly [23,24]. The immunogenicity of D15 has been demonstrated in both *Haemophilus ducreyi *[25] and *Pasteurella multocida *[26]. Thus, D15 is a highly conserved antigen and may be a useful component of a universal subunit vaccine against *Haemophilus *infection [27]. Subunit NqrA was identified as a 48-kDa outer membrane protein with immunogenic in serotype 1 or 5A and is common to 12 APP serotypes, but is not present in related Gram-negative swine pathogen species [12]. Outer membrane protein P5 (or its precursor), which is a homolog of OmpA in *Pasteurella trehalosi *and *Escherichia coli*, may be involved in the adherence of bacteria to nasopharyn-geal mucin [28].

The ribosomal subunit proteins L7/L12 (spot APr4) and pyruvate dehydrogenase subunit E1 (Spots APr10, 11) [29] were identified as immunogenic proteins in members of the *Bacillus cereus *group [30], but have not previously been identified as such in APP before.

The remaining 23 proteins identified in our study are being reported here as immunoreactive proteins for the first time, to our knowledge. The majority of these immunoreactive proteins were linked to housekeeping functions, such as energy production (e.g. spot APr8, APr9), carbohydrate transport and metabolism (e.g. spot APs2, APs13), amino acid transport and metabolism (e.g. APs19), and chaperones (e.g. spot APs3, APs5 ), reflecting their importance for survival.

We ultimately concluded that the spots labeled as APs6, APs9 and APr15, APr16 were likely mixtures of several proteins; there were no corresponding sequences for these spots available for Mascot searches. Our inability to identify these spots can be attributed to several reasons, including the genome sequence of APP serotype 1 being incomplete, the spots not being evident on duplicated gels, and/or a few adjacent spots being mixed together.

Highly conserved immunogenic proteins could potentially induce protection against a wide variety of bacterial strains and are thus attractive novel vaccine candidates. Goure et al. have identified APP genes that are conserved among all 15 serotypes by comparative genomic hybridization [31]. Of these conserved genes, the genes encoding protective surface antigen D15 and outer membrane protein P5 were observed in our results. Subunit NqrA was also demonstrated to be common to 12 serotypes by Cruz et al. [12]. It is notable that outer membrane protein APBr3 was also found in different APP serotypes and showed a high level of conservation across serotypes.

The reliability of our protein identification was further confirmed by comparison of the experimental MW and p*I *values of the protein spots on the 2DE gels with the theoretical ones. Overall, the majority of the theoretical and experimental values matched well, though some discrepancies remained. Similar migration for several proteins has previously been described in proteomic analysis of serotype 3 APP [20] and other pathogens [32,33]. The presence of natural isoforms, post-translational proteolytic processing and/or modification, or artifacts related to sample preparation might explain the discrepancies. In addition, 7 proteins were identified from more than one position on the gels, including outer membrane protein P5, perhaps due to the presence of horizontal isoforms.

Different western blot results were obtained with analyses from swine versus rabbit sera. Only one common protein in the two species (outer membrane protein P5) was identified. Further research is needed to illuminate the factors that may explain this divergence.

## Conclusions

We optimized 2DE sample preparation for APP, and obtained clearly visualized 2DE profiles with abundant spots. This method can applied in proteome studies of other Gram-negative bacteria. Using this approach, we identified 32 proteins by western blot analysis using convalescent sera from swine and hyperimmune sera from rabbits. The newly identified immunoreactive APP serotype 1 proteins are of great interest in terms of understanding pathogen-host interaction and can be considered novel vaccine candidates.

## Methods

### Strain and culture conditions

APP virulent strain S259 was purchased from the Control Institute of Veterinary Bioproducts and Pharmaceuticals of China. The cells were cultured in Trypticase Soy Broth (TSB, Merck, Germany); nicotinamide adenine dinucleotide (NAD, Sinopharm, Shanghai, China) was added to the medium at a final concentration of 0.01%. One-hundred-milliliter cultures were shaken overnight at 37°C on a rotary incubation shaker running at 180 rpm until they reached the late stage of exponential phase. Cells were harvested by centrifugation at 10,000 ×g for 10 min at 4°C and washed three times with phosphate buffered saline (PBS).

### Sera

Convalescent serum from swine naturally infected with APP serotype 1 were screened by two steps. Step 1, seeking convalescent sera against APP. A total of 769 swine serum samples were collected from 29 herds in different provinces of China in 2009. Then ApxIV-ELISA was performed to screen convalescent serum from naturally infections as reported previously [34]. The ApxIV-ELISA has the advantage over other serological tests that it does not show cross-reactions with other bacterial species and that it is highly sensitive and allows also the serological detection of pigs infected by the different serotypes of APP or carrying the agent without apparent clinical signs or symptoms of infection. Furthermore the test allows clear differentiation between pigs infected with APP and healthy pigs that were vaccinated against APP. In addition, we have traced the background of corresponding swine. These swine were never immunized any vaccines against APP infection and were apparently healthy. Step 2, seeking convalescent sera against APP serotype 1 with S259 whole cell ELISA as described elsewhere [18]. As negative controls, we tested sera from newly born piglets using the same methods.

To prepare hyperimmune serum, rabbits were immunized with formaldehyde-inactivated APP S259 vaccine, with Montanide ISA 206 VG (SEPPIC, France) used as an adjuvant. Two doses of 1.0 × 10^9 ^cells/rabbit were administered by intramuscular injections with a 3-week interval. Sera from negative control and immunized rabbits were collected before the first and after the second immunization. Serum titers were evaluated with S259 whole cell ELISA.

Our experimental research has been performed with the approval of Institute of Veterinary Medicine, Jiangsu Academy of Agricultural Sciences(SYXK 2010-0005)

### Protein sample extraction

Washed APP S259 cell pellets were re-suspended in 5-mL sample preparation solutions (7 M urea, 2 M thiourea, 4% w/v CHAPS, and 40 mM DTT) and sonicated in an ice bath for 50 cycles (5 s on, 10 s off) at a power setting of 200 W. The cell lysate was incubated for 30 min at 25°C to solubilize proteins (vortexing every 10 min) and centrifuged at 10,000 ×g for 20 min at 25°C to pellet the insoluble components. To precipitate proteins, the cleared supernatants were treated with pre-chilled 100% trichloroacetic acid (TCA) to a final concentration of 10% and incubated in ice water for 30 min. Precipitated protein was collected by centrifugation at 10,000 ×*g *for 10 min at 25°C and washed twice with pre-chilled acetone. The final pellet was air-dried.

### Isoelectric focusing (IEF)

The dried pellet was dissolved in sample preparation solution, then incubated for 30 min at 25°C (vortexing every 10 min) and centrifuged at 10,000 ×g for 20 min at 25°C. Before rehydration, the supernatant was treated with a 2-D Clean-up Kit (GE Healthcare, Shanghai, China) to remove contaminants that interfere with IEF. Then IEF was performed using the Ettan IPGphor 3 IEF system (GE Healthcare, Shanghai, China) with 13-cm (Immobiline DryStripk, pH 4-7; GE Healthcare, Shanghai, China) as well as 7-cm (Immobiline DryStrip, pH 7-11; GE Healthcare, Shanghai, China) immobilized pH gradient (IPG) gel strips. IPG strips were rehydrated overnight at room temperature with rehydration solution [7 M urea, 2 M thiourea, 2% w/vCHAPS, 0.2% w/vDTT, 0.5% v/vIPG buffer (same range as the IPG strip), and 0.002% w/v bromophenol blue]. Each of the 13-cm and 7-cm strips was loaded with 200 μg and 100 μg of protein, respectively. IEF was carried out at 20°C for 11.5 h (maximum voltage of 8,000 V, maximum current of 50 μA/IPG strip, total 28,000 Vh) with 13-cm IPG strips or for 5.5 h (maximum voltage of 5,000 V, maximum current of 50 μA/IPG strip, total 22,000 Vh) with 7-cm IPG strips, after 12 h of active rehydration.

### *Sodium dodecyl sulfate polyacrylamide gel electrophoresis *(*SDS-PAGE)*

Before running SDS-PAGE, each IPG strip was equilibrated for 15 min with 10 mg/ml DTT and 40 mg/ml iodoacetamide in equilibration buffer (6 M urea, 75 mM Tris-HCl pH 8.8, 29.3% v/v glycerol, 2% w/v SDS, 0.002% w/v bromophenol blue). Each IPG strip plus an SDS-PAGE molecular weight standard (Invitrogen, Shanghai, China) was loaded on a 12% polyacrylamide gel and sealed with 1% agarose. Electrophoresis was performed at 15°C at an initial voltage of 110 V for 30 min, followed by 220 V until the tracking dye reached the gel bottom. Pairs of gels were run simultaneously, one for Coomassie G-250 stain and the other for western blot analysis. Each IEF/SDS-PAGE experiment was repeated three times.

### Western blot analysis

Protein samples from SDS-PAGE gels were transferred onto PVDF membranes (GE Healthcare, Shanghai, China) using a semi-dry blotting apparatus (TE77, GE Healthcare, Shanghai, China) for 2 h at 0.65 mA/cm^2^. Membrane-bound proteins were detected by staining with Ponceau S. Briefly, the PVDF membranes were submerged in Ponceau S stain solution (0.1% w/v Ponceau S, 5% v/v acetic acid) with gentle agitation for 5 min. The membranes were washed several times with distilled water (dH_2_O) until the protein spots were visible; the Ponceau S-stained membranes were digitally scanned with a Umax scanner (TE77, GE Healthcare, Shanghai, China). Ponceau S stain was removed by rinsing the membranes in dH_2_O with gentle agitation. After removing Ponceau S, the membranes were blocked with 5% w/v skim milk in 50 mM Tris-HCl buffer (pH 7.4) containing 0.05% Tween 20 (TBST) for 2 h at room temperature. The blocked-membrane was then incubated with convalescent sera from swine or hyperimmune sera from rabbit at room temperature for 2 h (1:1000 dilution with blocking buffer) and then washed three times with TBST for 15 min per wash. The membranes were incubated with Staphylococcal protein A labeled with horseradish peroxidase (Boster, Wuhan, China) which has been widespread applied as one kind of broad-spectrum secondary antibodies of most mammalian species [35-38] at room temperature for 1 h (1:10000 dilution with blocking buffer), washed three times with TBST, and developed by adding 3,3'-Diaminobenzidine (Tiangen, Beijing, China) until the optimum color was obtained. For each sample, the western blot was repeated three times.

### Alignment of total and immunoreactive protein images

Digital images of PVDF membranes stained with Ponceau S and immunoblotting analysis with sera from swine and rabbit were aligned using the layer function of Photoshop CS (Adobe). The corresponding protein spots and blots on each image were aligned precisely. The Ponceau S-stained image was also compared with the duplicated gel.

### Matrix Assisted Laser Desorption Ionization Time-of-Flight Mass Spectrometry (MALDI-TOF MS) and database searches

Immunoreactive proteins in the western blots were identified and excised from a duplicate SDS-PAGE gels and sent to Nanjing Ji'ao BioTechnologies Co., Ltd for in-gel trypsin digestion and MALDI-TOF MS. PMF data were analyzed using the MASCOT server http://www.matrixscience.com. In MASCOT searches, protein scores > 83 were significant (p < 0.05). The protein scores as well as the original PMF data such as extent of sequence coverage, number of peptides matched were used to accept protein identifications. The remaining proteins with scores < 83 were either verified manually or rejected.

### Bioinformatics analysis

Sequences of the identified proteins were searched in the BLASTX server http://blast.ncbi.nlm.nih.gov/ to find homologous sequences and searched in the PSORT server http://www.psort.org/ to predict protein subcellular localization.

## Competing interests

The authors declare that they have no competing interests.

## Authors' contributions

WZ conceived the idea of immunoproteomic study, participated in its design, performed major portion of the data analysis, drafted manuscript; JS carried out protein preparation, 2DE, western blot analysis and contributed in the preparation of the manuscript. GL assisted in protein sample extraction and western blot analysis; FT prepared of hyperimmune serum; YL contributed to the bioinformatics analysis; ZZ carried out ELISA; YW and ZW participated in the design, implementation, and coordination of the study; CL and HY reviewed the design plan, participated in revision of the final version of the manuscript. All authors read and approved the final manuscript.

## Supplementary Material

Additional file 1 **Figure S1. Comparison of western blot analysis with swine convalescent sera and ponceaus S stain, as an intermediate state, at pH 4-7**. In Photoshop, the immunoblot was used as the background layer and ponceaus S stain as the surface layer. The 0%, 20%, 40%, 60%, 80% and 100% transparency data are shown in **A**, **B**, **C**, **D**, **E **and **F**, respectively. Click here for file

Additional file 2 **Figure S2, Comparison of western blot analysis with rabbit hyperimmune sera and ponceaus S stain, as an intermediate state, at pH 4-7**. In Photoshop, the immunoblot was used as the background layer and ponceaus S stain as the surface layer. The 0%, 20%, 40%, 60%, 80% and 100% transparency data are shown in **A**, **B**, **C**, **D**, **E **and **F**, respectively. Click here for file

Additional file 3 **Figure S3. Comparison of western blot analysis with swine convalescent sera and ponceaus S stain, as an intermediate state, at pH 7-11**. In Photoshop, the immunoblot was used as the background layer and ponceaus S stain as the surface layer. The 0%, 20%, 40%, 60%, 80% and 100% transparency data are shown in **A**, **B**, **C**, **D**, **E **and **F**, respectively. Click here for file

Additional file 4 **Figure S4. Comparison of western blot analysis with rabbit hyperimmune sera and ponceaus S stain, as an intermediate state, at pH 7-11**. In Photoshop, the immunoblot was used as the background layer and ponceaus S stain as the surface layer. The 0%, 20%, 40%, 60%, 80% and 100% transparency data are shown in **A**, **B**, **C**, **D**, **E **and **F**, respectively. Click here for file
