# Supplementary figures and images for: Immunoproteomic analysis of bacterial proteins of Actinobacillus pleuropneumoniae serotype 1
Source: Proteome Sci. 2011 Jun 26;9:32. doi: 10.1186/1477-5956-9-32 (PMC3148531; doi:10.1186/1477-5956-9-32)

## Slide 1
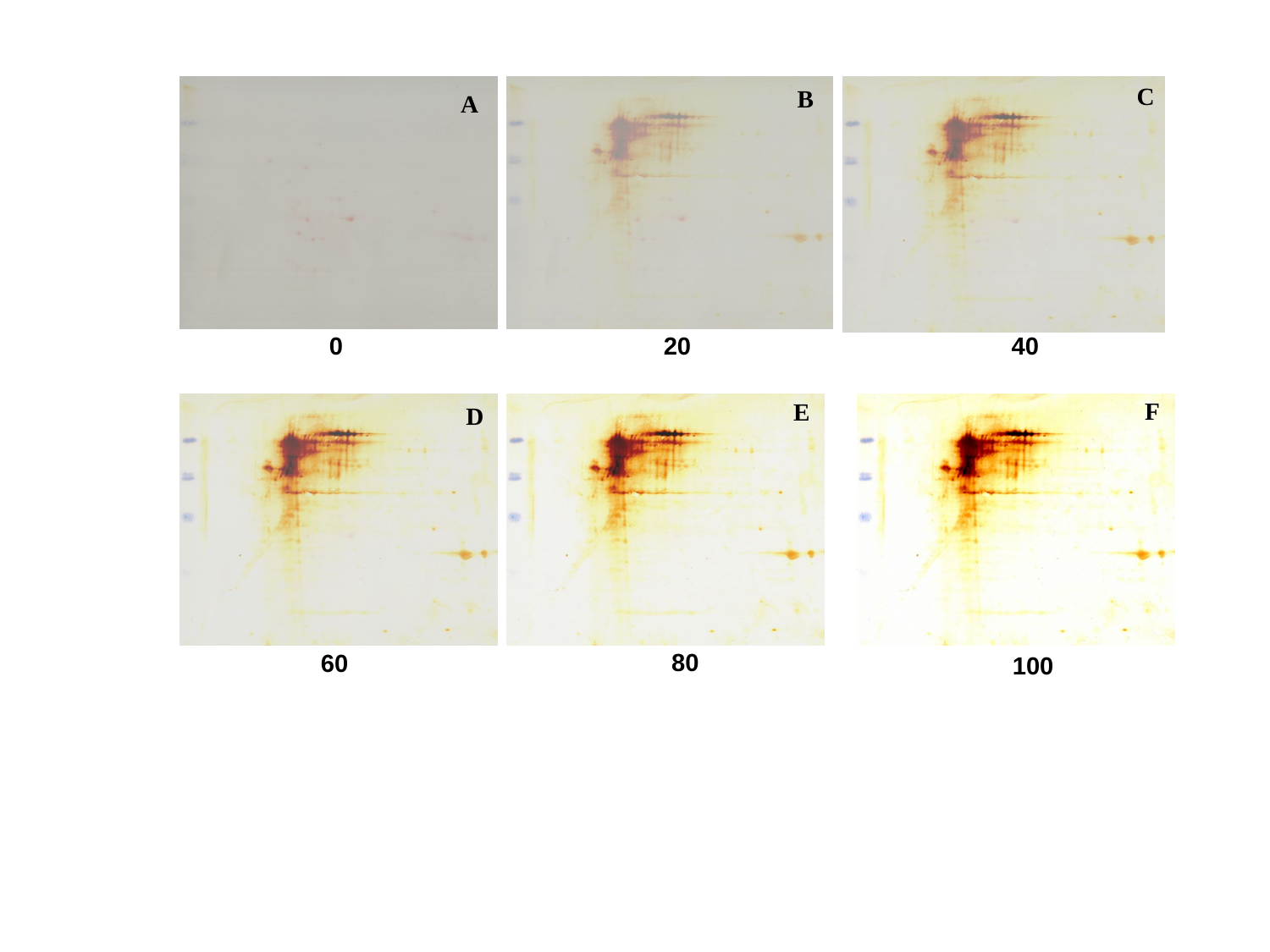

C
B
A
0
20
40
F
E
D
80
60
100

Supplement: Additional file 1 — Figure S1. Comparison of western blot analysis with swine convalescent sera and ponceaus S stain, as an intermediate state, at pH 4-7. In Photoshop, the immunoblot was used as the background layer and ponceaus S stain as the surface layer. The 0%, 20%, 40%, 60%, 80% and 100% transparency data are shown in A, B, C, D, E and F, respectively. [file 1477-5956-9-32-S1.PPT]

## Slide 1
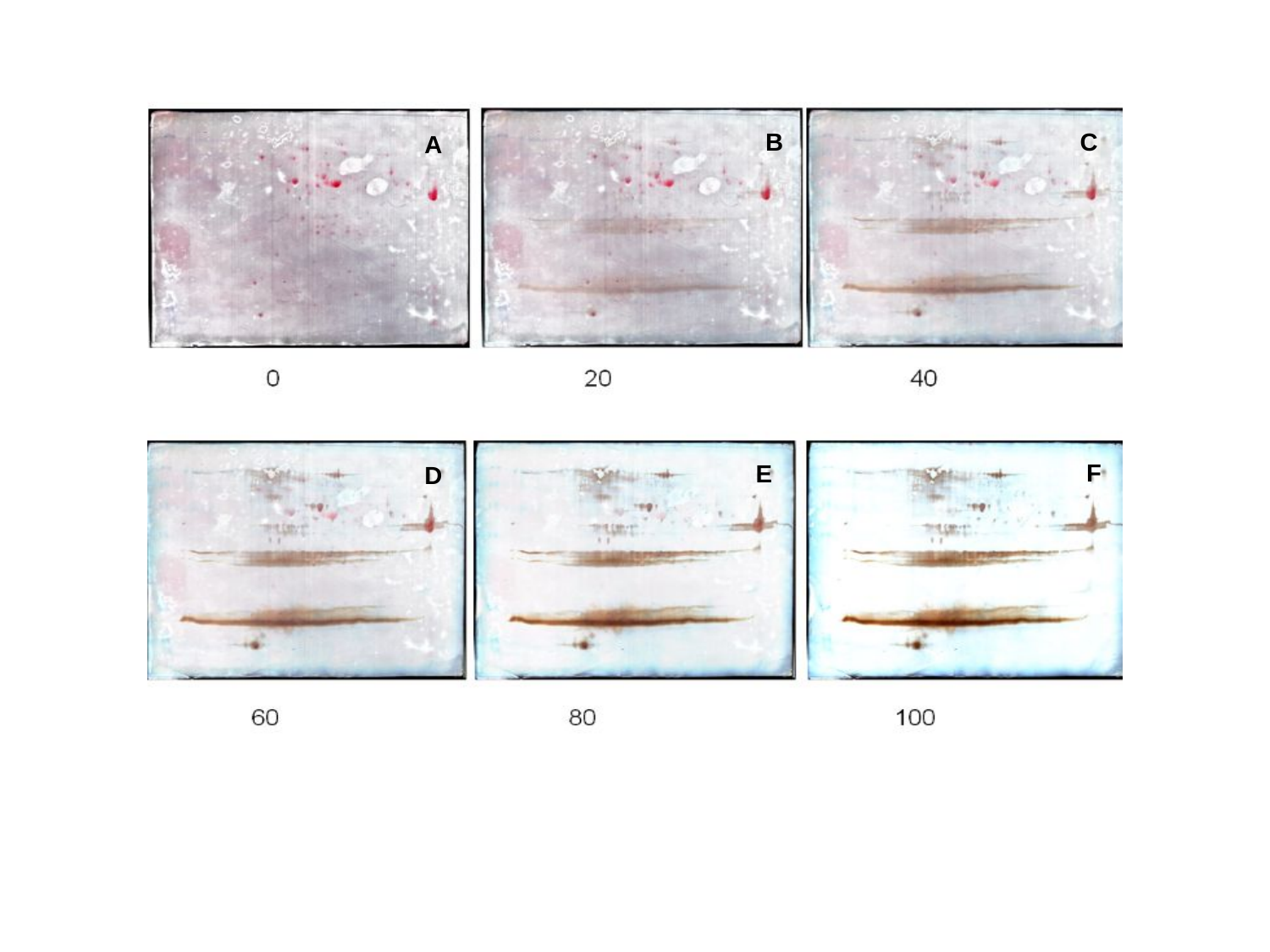

C
B
A
F
E
D

Supplement: Additional file 2 — Figure S2, Comparison of western blot analysis with rabbit hyperimmune sera and ponceaus S stain, as an intermediate state, at pH 4-7. In Photoshop, the immunoblot was used as the background layer and ponceaus S stain as the surface layer. The 0%, 20%, 40%, 60%, 80% and 100% transparency data are shown in A, B, C, D, E and F, respectively. [file 1477-5956-9-32-S2.PPT]

## Slide 1
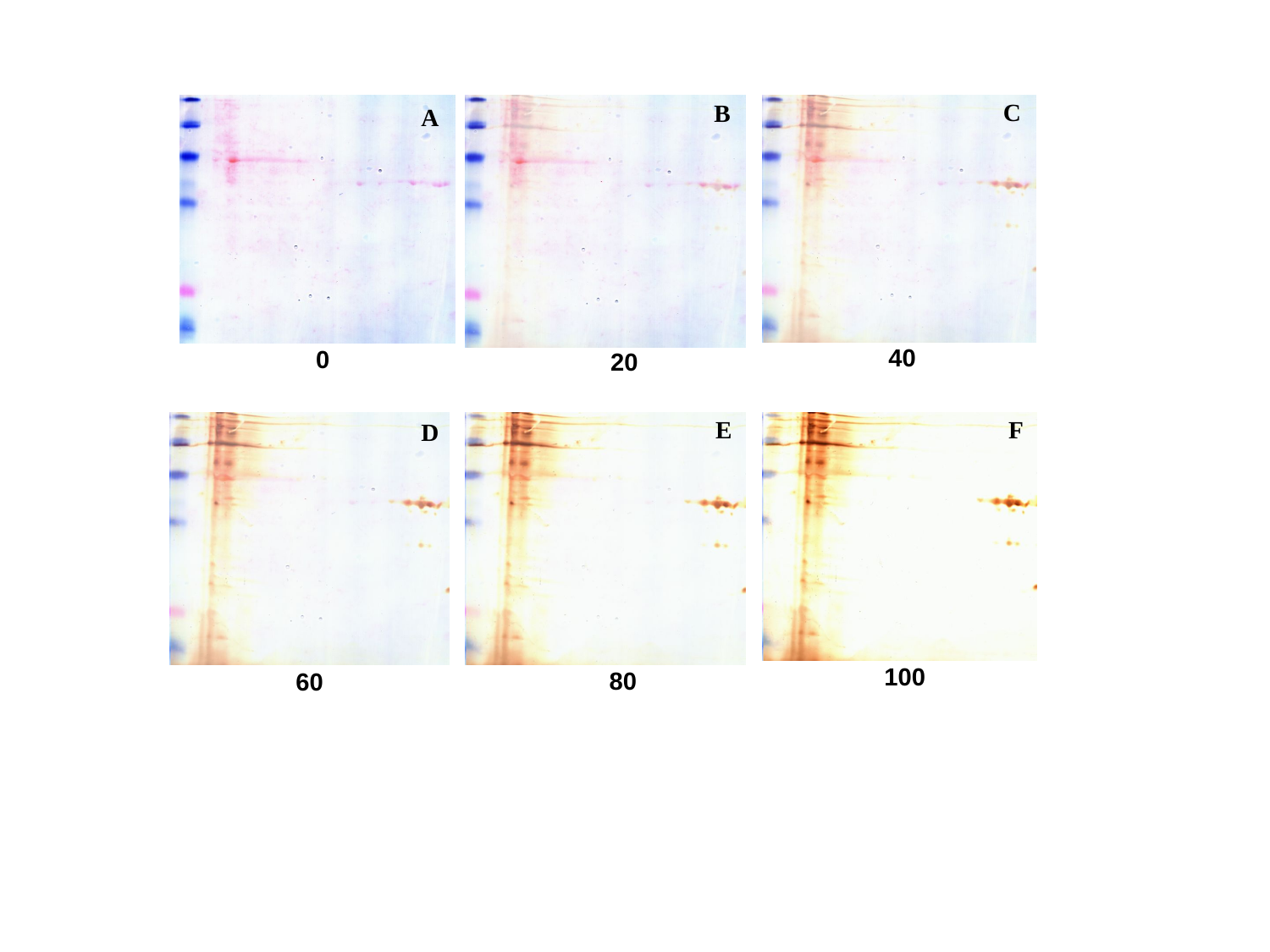

C
B
A
40
0
20
E
F
D
100
80
60

Supplement: Additional file 3 — Figure S3. Comparison of western blot analysis with swine convalescent sera and ponceaus S stain, as an intermediate state, at pH 7-11. In Photoshop, the immunoblot was used as the background layer and ponceaus S stain as the surface layer. The 0%, 20%, 40%, 60%, 80% and 100% transparency data are shown in A, B, C, D, E and F, respectively. [file 1477-5956-9-32-S3.PPT]

## Slide 1
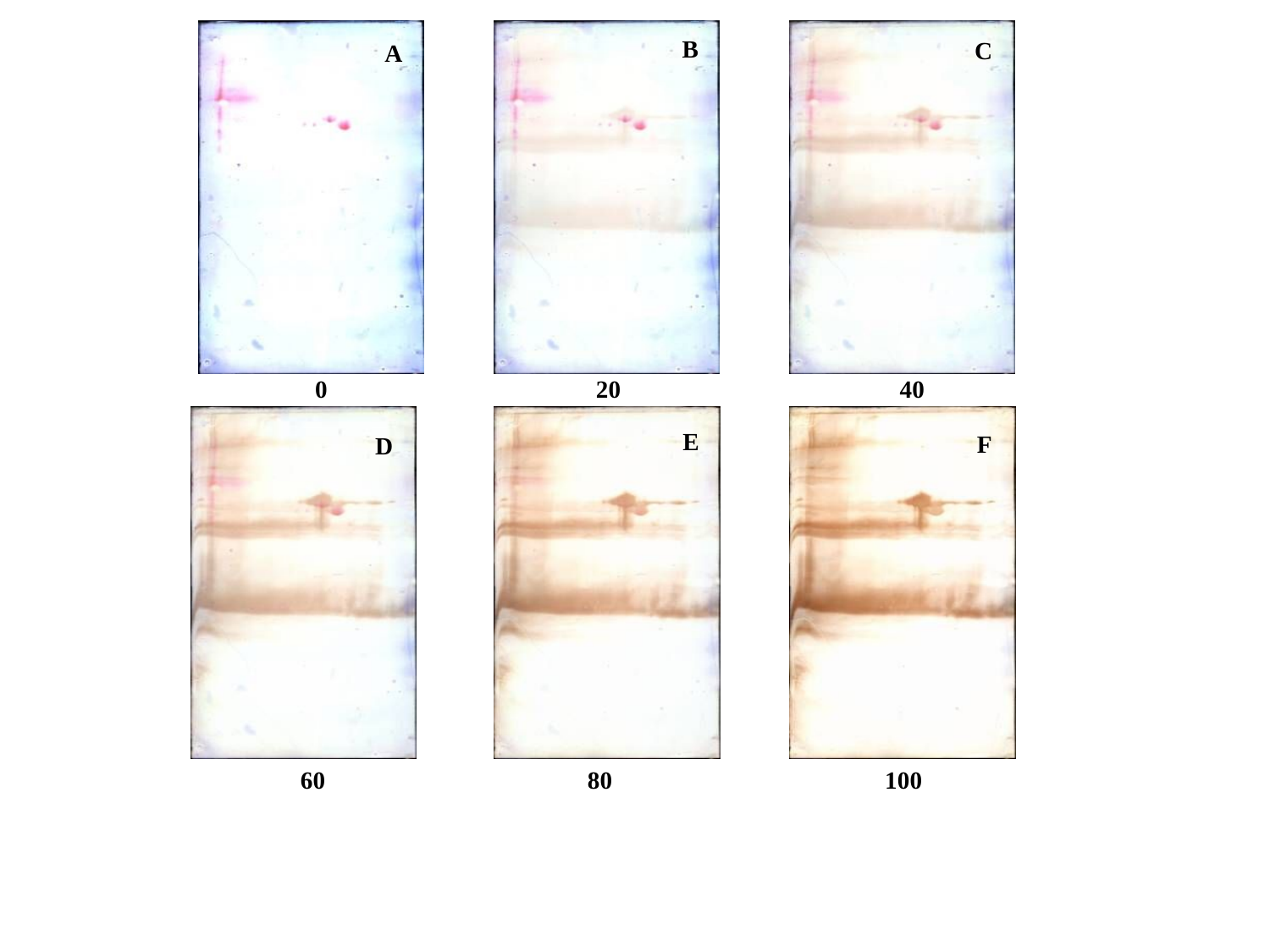

B
C
A
0
20
40
E
F
D
60
80
100

Supplement: Additional file 4 — Figure S4. Comparison of western blot analysis with rabbit hyperimmune sera and ponceaus S stain, as an intermediate state, at pH 7-11. In Photoshop, the immunoblot was used as the background layer and ponceaus S stain as the surface layer. The 0%, 20%, 40%, 60%, 80% and 100% transparency data are shown in A, B, C, D, E and F, respectively. [file 1477-5956-9-32-S4.PPT]
